# Surfactant-assisted Nanocasting Route for Synthesis of Highly Ordered Mesoporous Graphitic Carbon and Its Application in CO_2_ Adsorption

**DOI:** 10.1038/srep26673

**Published:** 2016-05-25

**Authors:** Yangang Wang, Xia Bai, Fei Wang, Hengfei Qin, Chaochuang Yin, Shifei Kang, Xi Li, Yuanhui Zuo, Lifeng Cui

**Affiliations:** 1 Department of Environmental Science and Engineering, University of Shanghai for Science and Technology, Shanghai, 200093, China; 2 Department of Environmental Science and Engineering, Fudan University, Shanghai 200433, China

## Abstract

Highly ordered mesoporous graphitic carbon was synthesized from a simple surfactant-assisted nanocasting route, in which ordered mesoporous silica SBA-15 maintaining its triblock copolymer surfactant was used as a hard template and natural soybean oil (SBO) as a carbon precursor. The hydrophobic domain of the surfactant assisted SBO in infiltration into the template’s mesoporous channels. After the silica template was carbonized and removed, a higher yield of highly-ordered graphitic mesoporous carbon with rod-like morphology was obtained. Because of the improved structural ordering, the mesoporous carbon after amine modification could adsorb more CO_2_ compared with the amine-functionalized carbon prepared without the assistance of surfactant.

Much attention has paid to the preparation and characterization of ordered mesoporous carbons (OMCs) in recent years. They can be used potential adsorbents, catalyst supports, hydrogen storage materials, and electrode materials owing to their special structure-dependent properties, such as highly uniform pore structure, high specific surface area (SSA), and large pore volume^1^,^2^,^3^,^4^.

Compared to other porous materials such as zeolites^5^ and metal-organic frameworks (MOFs)^6^, OMCs can be highly promising in CO_2_ adsorption due to their unique surface property and wide availability. The higher mesostructural ordering property generally permits the higher surface chemical modification potential for OMCs^7^.

So far, OMCs are mainly synthesized through two methods. First, the traditional chemical soft-template method is extensively applied to prepare ordered mesoporous silicas (OMSs)^8^. However, this method has high requirements on the synthetic conditions because it requires the use of a phenolic-resin-based carbon precursor^9^,^10^,^11^. The other one, the hard-template method (i.e. nanocasting route), is more frequently used and adopts OMSs as sacrificial scaffolds, in which a carbon precursor is fabricated within the pore channels of OMSs, and then the silica template is carbonized and selectively removed. Based on this method, a multitude of OMCs with diversity in mesostructure, pore sizes or even graphitic characteristics can be prepared^12^,^13^,^14^,^15^. Nevertheless, to improve the mesostructural ordering and the yield of carbon materials, operators have to include an inconvenient and time-consuming impregnation step in this route.

It is well-known that the sorption and electrical properties of OMCs are closely related to their graphite nature. Until now, several synthetic approaches that are based on the hard template method have been reported in the literatures for the graphitization OMCs: (1) high-temperature treatments (>2000 °C) to induce the graphitization of OMCs, but often sacrificing structural ordering^16^,^17^, (2) using graphitizable polyaromatic compounds as carbon precursors to get graphitic carbon at relative lowtemperature^18^,^19^. However, this route usually involves a multi-step wet impregnation procedure for the pore infiltration assisted by an organic solvent, which can result in low levels of infiltration because of the competition of the solvent^13^. (3) synthesis by chemical vapor deposition (CVD) method at high temperature (>900 °C)^5^,^20^,^21^. Nevertheless, the yield of the obtained carbon materials by CVD method without surface treatment of the template is very low^22^. As reported, ordered mesoporous graphitic carbon can be prepared by a one-step solid-state method using metal phthalocyanines (MPc) and SBA-15, this synthesis is strategically combined with *in-situ* CVD and catalytic graphitization^7^. In our previous study, a simple solid- liquid grinding/templating route using cheap natural seed fat (e.g. soybean oil SBO) as a carbon precursor was applied to synthesize OMC with graphitic frameworks^23^. However, we did not largely improve the yield and structural ordering of the carbon materials, because the interaction between organic seed fat and surface hydroxyl groups of the silica template was very weak.

Herein, we demonstrate a facile “surfactant-assisted” nanocasting route for the synthesis of highly ordered mesoporous graphitic carbon with improved yield. This synthesis was achieved using as-prepared OMS without removal of P123 surfactant as the hard template, natural organic soybean oil (SBO) as a carbon precursor which can be easily infiltrated into the mesopore channels of the silica template with the help of the hydrophobic domain of the triblock copolymers to reach a high filling. The structural order and textural properties of the obtained carbon materials were characterized by X-ray diffractometry (XRD), thermogravimetric (TG) analysis, nitrogen adsorption-desorption, Raman spectroscopy, scanning electron microscopy (SEM) and transmission electron microscopy (TEM). Meanwhile, a CO_2_ adsorption capacity on the amine- modified mesoporous carbon materials with different textural structures was investigated and compared.

## Methods

### Materials preparation

Mesoporous silica SBA-15 template with rod-like morphology was synthesized as the reported procedure except enlarging the amount by ten times^24^. Highly ordered mesoporous graphitic carbon was synthesized using one-step “surfactant-assisted” nanocasting route without the help of any organic solvent. In a typical synthesis, the OMS as-prepared, SBA-15 template (without removal of self-possessed P123 surfactant, denoted as SBA-15-1), was dispersed into the liquid state SBO and stirred for 5–8 h at room temperature, during this process the SBO molecules can be easily adsorbed by the mesoporous silica template with the help of the hydrophobic domain of the triblock copolymers. After filtering, the SBO/SBA-15-1 composite was transferred into a tube furnace to carbonize the precursor at 900 °C under nitrogen atmosphere for 4 h to obtain a graphitic carbon-silica composite (GC/SBA-15-1). The resultant ordered mesoporous graphitic carbon referred to as MGC-1 can be acquired after removal of the silica template with 10% HF aqueous solution.

For comparison, another mesoporous graphitic carbon was prepared using calcined mesoporous SBA-15 (without surfactant in mesochannels, named as SBA-15-2) as the hard template by the same method and denoted as MGC-2.

### Surface modification with amine groups

Amine-modified carbon materials (MGC-1 and MGC-2) were prepared according to the procedure reported previously^25^. Typically, 1.0 g of MGC-1 or MGC-2 was soaked in a 10 g methanol solution containing 0.67 g TEPA at room temperature for 3 h, then dried at 45 °C under reduced pressure overnight to evaporate methanol and avoid amino group degradation. The resulting hybrid materials were designated as TEPA-MGC-1 and TEPA-MGC-2, respectively, and the TEPA weight percentage in the hybrid materials was about 40%.

### Characterization

Small-angle XRD patterns were recorded on a Rigaku D/MAX-2550VB/PC diffractometer under the following conditions: θ-2θ mode, CuKα_1_ radiation (λ = 1.5406 Å), 40 kV, 200 mA, and scanning step 0.02°/sec. Wide-angle XRD patterns were recorded in the same mode, except the condition at 100 mA. Raman spectra were recorded with a Dilor LabRam-1B RS device at the He-Ne laser (excitation wavelength = 632.8 nm). SEM images were measured on a Philips XL-30 SEM device at an acceleration voltage of 25 kV. TEM images were taken on a JEOL JEM-2010 TEM device with an acceleration voltage of 200 kV. After the samples were vacuum-degassed at 200 °C for 6 h, nitrogen sorption isotherms were recorded on a BeiShiDe 3H-2000PS4 device at −196 °C. Moreover, the Brumauer–Emmett–Teller (BET) SSAs were calculated. From the desorption branches of the isotherms, pore size distributions were deduced via the Barrett– Joyner–Halenda (BJH) method. Total pore volume (V_t_) was determined at a relative pressure of 0.98. TG analysis was carried out using a PerkinElmer STA-8000 analyzer (America) from 25 to 800 °C, in a 25 mL min^−1^ air flow and at a heating rate of 10 K min^−1^.

### CO_2_ adsorption analysis

The CO_2_ adsorption isotherms of the samples were measured by a simultaneous DSC-TGA analysis using a STA-8000 instrument under ambient pressure (1.0 atm). In a typical procedure, the sample was degassed at 100 °C for 60 min under an Ar flow to remove physisorbed moisture and impurities adsorbed, after the temperature was decreased to 75 °C, the sample was allowed to adsorbed CO_2_ by passing CO_2_ at a flow rate of 25 mL min^−1^. Upon introduction of the CO_2_ gas, a weight gain was observed due to CO_2_ physical absorption on the sample surface, the process was continued for 90 min.

## Results and Discussion

**Figure 1:**
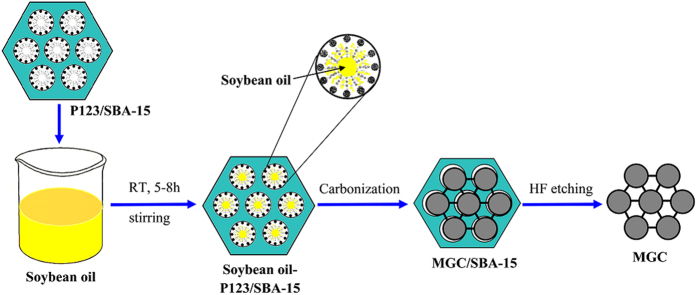
Schematic illustration of the synthesis process of ordered mesoporous graphitic carbon by “surfactant-assisted” nanocasting route.

Figure 1 shows the synthesis process of this “surfactant-assisted” nanocasting route for the ordered mesoporous graphitic carbon. The main advantage of this method is that the organic surfactant P123(EO_20_PO_70_EO_20_) in SBA-15 template does not need to be removed since it can effectively adsorb the organic liquid carbon precursor in mesochannels of the template and finally effectively enhance the yield of the graphitic carbon materials.

**Figure 2:**
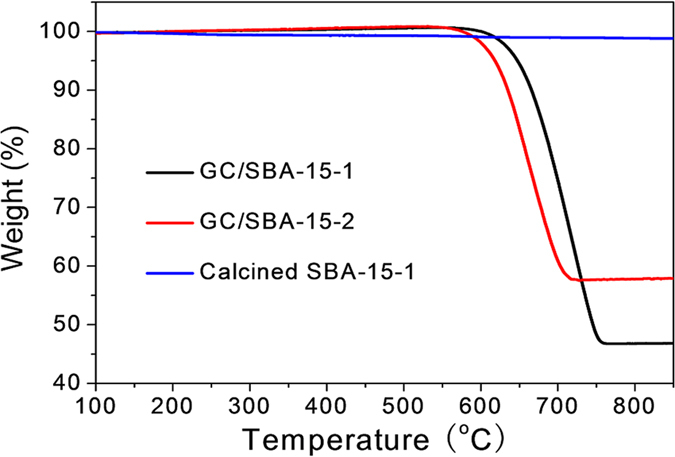
TG curves of different carbon-silica composites.

To demonstrate that this innovative synthetic route can efficiently help to promote the inclusion of SBO molecules in SBA-15 mesochannels, we prepared the graphitic carbon-silica composites (GC/SBA-15-1 and GC/SBA-15-2) using two different SBA-15 templates and subjected to TG analysis to ascertain the amount of graphitic carbon loaded on SBA-15 via the complete combustion of the carbon at high temperature. As showed in Fig. 2, the mass loss increases with temperature rise, the final weight losses of GC/SBA-15-1 and GC/SBA-15-2 were calculated to be 53.3% and 42.4%, respectively, suggesting that more SBO molecules are efficiently included in SBA-15-1 template via this “surfactant-assisted” strategy. To exclude the increased carbon content derived from P123 in GC/SBA-15-1, we set the mesoporous silica template SBA-15-1 as-prepared (without removal of surfactant) to carbonization (the obtained composite named as C/SBA-15-1) and further analyzed by TG (Fig. 2). Clearly, the residual carbon content in C/SBA-15-1 is only 1.1 wt%, which is much lower than enhanced carbon content (10.9 wt%) between GC/SBA-15-1 and GC/SBA-15-2.

**Figure 3:**
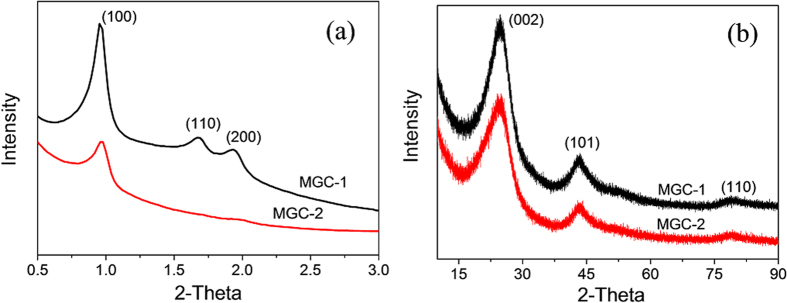
(**a**) Low-angle XRD patterns and (**b**) wide-angle XRD patterns of MGC-1 and MGC-2.

**Figure 4:**
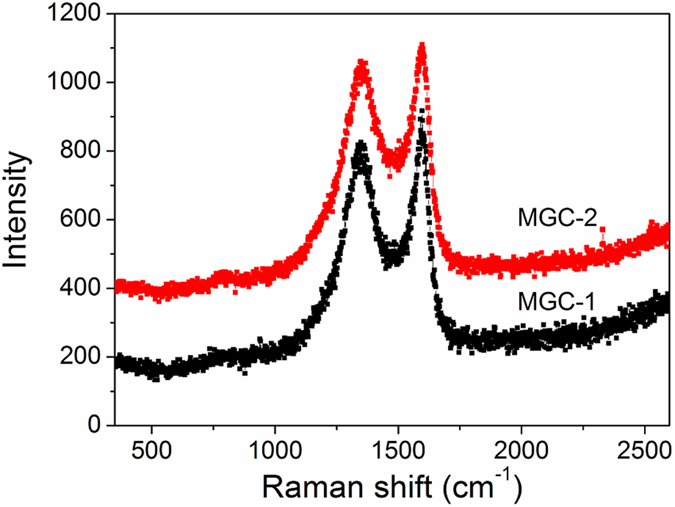
Raman spectra of MGC-1 and MGC-2.

The silica templates were then removed from GC/SBA-15 by etching with an HF solution, followed by characterization via XRD. As showed on the low-angle XRD patterns (Fig. 3a), MGC-1 has three evident XRD peaks within 2*θ* = 0.8–2.1°, which are assigned to (100), (110) and (200) reflections of the 2D hexagonal (*p*6*mm*) symmetry, respectively. These results suggest MGC-1 is exactly a reverse duplication of the silica template, which are later proved by TEM. However, MGC-2 has only one lower-intensity peak corresponding to the (100) reflection, suggesting its mesostructural ordering of 2D hexagonal frameworks is lower compared with MGC-1. As showed on the wide-angle XRD patterns (Fig. 3b), both MGC-1 and MGC-2 have three characteristic peaks at around 25, 44, and 78°, corresponding to the (002), (101) and (110) reflections of graphitic carbons, respectively. Moreover, their diffraction intensities and peak widths are consistent with those of graphitic mesoporous carbon prepared by using aromatic compounds as carbon precursors^13^,^18^. The d-spacing values obtained from the (002) peak of MGC-1 and MGC-2 are 0.345 nm and 0.347 nm, respectively, both values are very close and slightly larger than that of pure graphite (0.335 nm). The Raman spectra of the MGC-1 and MGC-2 are shown in Fig. 4, both exhibit similar features with two main peaks at around 1330 cm^−1^ (D band) and 1580 cm^−1^ (G band), where the relatively low *I*_D_/*I*_G_ values (about 0.87 and 0.86 for MGC-1 and MGC-2, respectively) combined with a sharp G-band confirm that the obtained carbon materials have a moderate graphitization^13^,^26^, which can be confirmed by their TEM images (see below).

**Figure 5:**
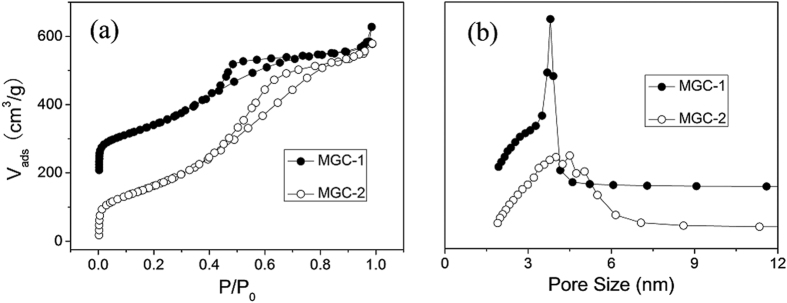
(**a**) N_2_ adsorption-desorption isotherms and (**b**) corresponding pore size distribution curves of the MGC-1 and MGC-2 samples.

As shown in Fig. 5, the nitrogen sorption isotherms of both MGC-1 and MGC-2 belong to the typical type IV showing a H1-type hysteresis loop within *P/P*_0_ of 0.4–0.8, which is typical of mesoporous non-siliceous materials prepared by the hard template method. However, the steep decrease of the nitrogen desorption in MGC-2 occurred at *P/P*_0_ of 0.45–0.65, indicating a broader pore size distribution compared with MGC-1, as confirmed by the pore size distribution curve in Fig. 5b. All the calculated textural parameters were listed in Table 1. The BET surface areas of MGC-1 and MGC-2 are 550.5 and 614.6 m^2^ g^−1^, respectively. The slight higher surface area, broader pore size distribution and higher pore volume for MGC-2 can be ascribed to the incompletely filling of carbon precursor in the channels of silica template. After amine-modification, the BET surface areas of TEPA-MGC-1 and TEPA-MGC-2 reduced significantly, as well as their pore volume. The elemental analysis results in Table 1 reveal that the amine groups have been grafted on the surface of carbon materials, and the TEPA contents (wt.%) according to calculation in the resulting hybrid materials are very close to the initial added weight percentage. It is should be noticed that the C/N ratio of TEPA-MGC-1 is slightly lower than that of TEPA-MGC-2, indicating a richer amine groups in the TEPA-MGC-1. This suggested that the highly ordered mesostructure plays a dominant role compared with surface area and pore volume for surface modification of MGC materials.

**Figure 6:**
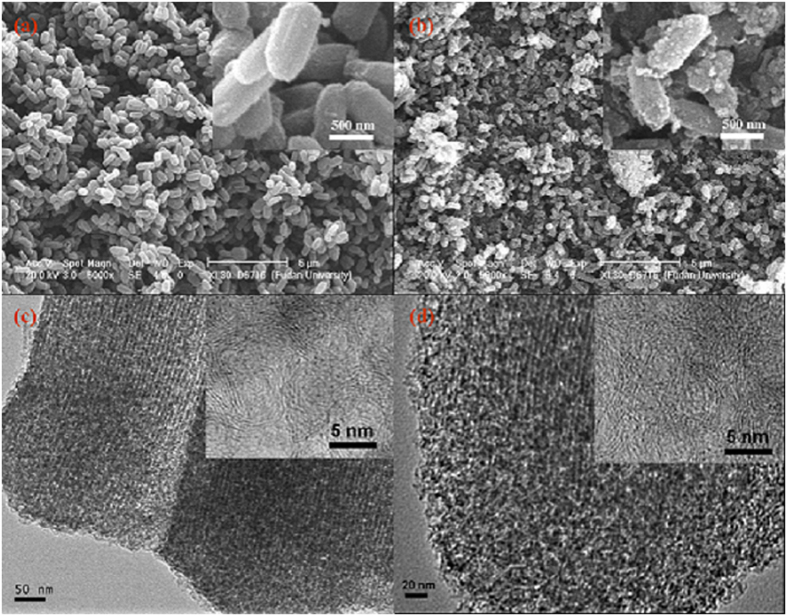
Typical SEM and TEM images of (**a**,**c**) MGC-1 and (**b**,**d**) MGC-2.

The morphology and textural structure of the MGC-1 and MGC-2 were investigated by using SEM and TEM. In Fig. 6a, it can be seen that MGC-1 has a well-defined rodlike morphology with regular surface after removing the silica template, suggesting that this carbon material was an exact reverse-replica of the SBA-15 template. While a serious large-scale texture deformation is observed for MGC-2, even to the rough surface residues in a disorderly fashion (Fig. 6b inset). The improved textural framework for MGC-1 is further evidenced by the TEM images in Fig. 6c, it can be found that the linear arrays of mesochannels along [100] direction are observed as arranged in an orderly, regular pattern. Whereas a much less organized mesoporous structure with a considerable number of defects is detected for MGC-2, indicating producing larger mesoporous channels which is in agreement with the result of pore size distribution curve as shown in Fig. 5b. As showed on the high-magnification TEM images, the irregular orientation of graphite layers is evidently stacked for both samples, which indicates the appearance of partially-graphitized carbon frameworks.

**Figure 7:**
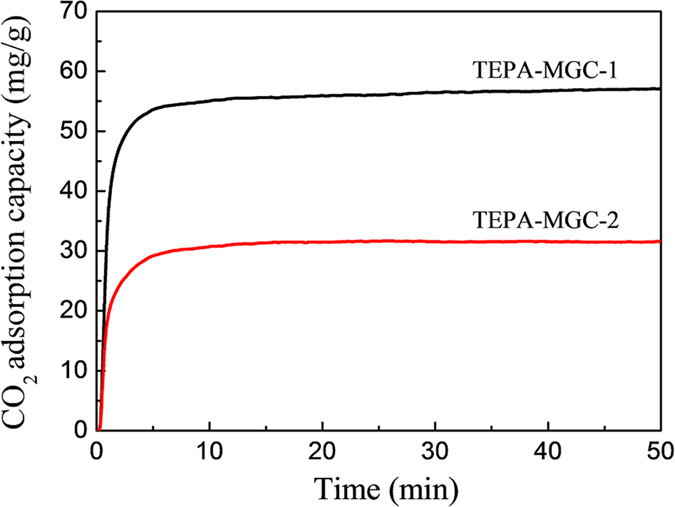
CO_2_ adsorption isotherms at 75 °C of the TEPA-MGC-1and TEPA-MGC-2.

The enormous emission amount of CO_2_ into atmosphere by human activity has caused serious greenhouse effect and global warming^27^. CO_2_ capture and storage is recognized as a major technology to reduce CO_2_ emissions and combat climate change. It has been reported that amine-functionalized mesoporous materials can function as effective solid absorbents for CO_2_ capture and separation^28^,^29^,^30^. Herein, CO_2_ adsorption capacity of TEPA-modified MGC-1 and MGC-2 was investigated and compared on a thermogravimetric analyzer at the temperature of 75 °C. Figure 7 shows the CO_2_ adsorption isotherms under ambient pressure (1.0 atm). Clearly, the adsorption equilibration of both samples is achieved within a short time, and the CO_2_ adsorption capacity for the TEPA-MGC-1 (57.2 mg g^−1^) has a distinct advantage over TEPA-MGC-2 (31.3 mg g^−1^). This higher adsorption performance for the TEPA-MGC-1 should be attributed to the improved order of mesochannels and the presence of fewer textural structure defects. The improved textural structure, especially the well-organized 2D hexagonal frameworks facilitate the dispersion of amine group on the mesoporous carbon support, and further promote CO_2_ adsorption due to the increasing diffusion of gas molecular in the ordered mesopore channels.

## Conclusions

A simple surfactant-assisted nanocasting route using natural soybean oil as a carbon precursor was designed to synthesize highly-ordered mesoporous graphitic carbon. XRD, TG, Raman, nitrogen sorption, SEM and TEM show that the precursor was easily infiltrated into the mesopore channels of the silica template, with the help of the hydrophobic domain of the self-possessed triblock copolymers. Also this mesoporous carbon has much higher yield and structural ordering than the carbon material prepared from the surfactant-free route. Because of this improvement, the mesoporous graphitic carbon after amine modification could absorb more CO_2_ capacity under ambient pressure.

## Additional Information

**How to cite this article**: Wang, Y. *et al.* Surfactant-assisted Nanocasting Route for Synthesis of Highly Ordered Mesoporous Graphitic Carbon and Its Application in CO_2_ Adsorption. *Sci. Rep.* **6**, 26673; doi: 10.1038/srep26673 (2016).

## Figures and Tables

**Table 1:** Textural parameters of the MGC-1, MGC-2, and their amine-modified samples.

Samples	S_BET_ (m^2^/g)	Pore size (nm)	Pore volume (cm^3^/g)	d_002_ (nm)	Elemental analysis		
					C (wt. %)	N (wt. %)	H (wt. %)
MGC-1	550.5	3.5	0.772	0.345	98.12	0.96	0.92
MGC-2	614.6	4.2	1.122	0.347	97.87	1.22	0.91
TEPA-MGC-1	7.7	3.78	0.215	/	79.92	15.14	4.94
TEPA-MGC-2	8.9	4.27	0.180	/	80.75	14.49	4.76
